# Low-phytate wholegrain bread instead of high-phytate wholegrain bread in a total diet context did not improve iron status of healthy Swedish females: a 12-week, randomized, parallel-design intervention study

**DOI:** 10.1007/s00394-018-1722-1

**Published:** 2018-05-23

**Authors:** Michael Hoppe, Alastair B. Ross, Cecilia Svelander, Ann-Sofie Sandberg, Lena Hulthén

**Affiliations:** 1 Department of Gastroenterology and Hepatology, Clinical Nutrition Unit, Sahlgrenska University Hospital, Gothenburg, Sweden; 2 Department of Internal Medicine and Clinical Nutrition, Sahlgrenska Academy at the University of Gothenburg, Gothenburg, Sweden; 3 Department of Biology and Biological Engineering, Food and Nutrition Science, Chalmers University of Technology, Gothenburg, Sweden

**Keywords:** Non-heme iron, Iron status, Phytate, Wholegrain, Dietary intervention, Women

## Abstract

**Purpose:**

To investigate the effects of eating wholegrain rye bread with high or low amounts of phytate on iron status in women under free-living conditions.

**Methods:**

In this 12-week, randomized, parallel-design intervention study, 102 females were allocated into two groups, a high-phytate-bread group or a low-phytate-bread group. These two groups were administered: 200 g of blanched wholegrain rye bread/day, or 200 g dephytinized wholegrain rye bread/day. The bread was administered in addition to their habitual daily diet. Iron status biomarkers and plasma alkylresorcinols were analyzed at baseline and post-intervention.

**Results:**

Fifty-five females completed the study. In the high-phytate-bread group (*n* = 31) there was no change in any of the iron status biomarkers after 12 weeks of intervention (*p* > 0.05). In the low-phytate bread group (*n* = 24) there were significant decreases in both ferritin (mean = 12%; from 32 ± 7 to 27 ± 6 µg/L, geometric mean ± SEM, *p* < 0.018) and total body iron (mean = 12%; from 6.9 ± 1.4 to 5.4 ± 1.1 mg/kg, *p* < 0.035). Plasma alkylresorcinols indicated that most subjects complied with the intervention.

**Conclusions:**

In Swedish females of reproductive age, 12 weeks of high-phytate wholegrain bread consumption had no effect on iron status. However, consumption of low-phytate wholegrain bread for 12 weeks resulted in a reduction of markers of iron status. Although single-meal studies clearly show an increase in iron bioavailability from dephytinization of cereals, medium-term consumption of reduced phytate bread under free-living conditions suggests that this strategy does not work to improve iron status in healthy women of reproductive age.

## Introduction

Iron deficiency anemia reflects a functional iron deficiency that can lead to impaired work performance [1], altered cognitive function [2], impaired immunity [3], and increased risk of maternal and child mortality [4–6]. Iron deficiency and iron deficiency anemia is a major global health problem, especially for women of childbearing age [7].

Absorption is the primary mechanism by which iron balance is controlled in healthy individuals. Only a fraction of non-heme iron is absorbed and its availability is profoundly influenced by various inhibitors and enhancers [8]. The net absorption of total iron from the diet is a balance between the effects of these enhancers and inhibitors. A potent inhibiting factor, even at very low concentrations, is phytate and some of its degradation products [9, 10]. The main source of phytate in the diet is usually from cereal products such as bread and correlates with the fiber content in cereals. Thus, diets rich in whole grains and dietary fiber can result in a reduced bioavailability of dietary non-heme iron mainly due to the concomitant increased intake of phytate. Due to the substantial beneficial effects from whole grains on preventing certain lifestyle diseases “Dietary Guidelines for Americans” recommends a diet where at least half of the recommended 6-ounce equivalents of grain per day at the 2000-calorie level, should be wholegrain [11]. It is also recommended that the daily fiber intake is 14 g/1000 kcal, i.e. 28–34 g/day, depending on age and gender [11]. However, since a high intake of wholegrain fiber-rich bread can result in a decreased iron status of women with otherwise sufficient iron stores [12] these recommendations may be unfavorable for maintaining adequate iron status, especially in women of fertile age.

Previously it has been hypothesized that reducing phytate content of plant foods containing iron would improve bioavailability, and from this improve iron status. Single-meal studies have demonstrated that reduction of phytate using sourdough fermentation increases iron bioavailability [13]. Here we have used a randomized, double-blind, parallel-design intervention study to test the hypothesis that wholegrain rye bread with very low amounts of phytate would improve markers of iron status as compared to wholegrain rye bread with high amounts of phytate.

## Subjects and methods

### Study design

**Fig. 1:**
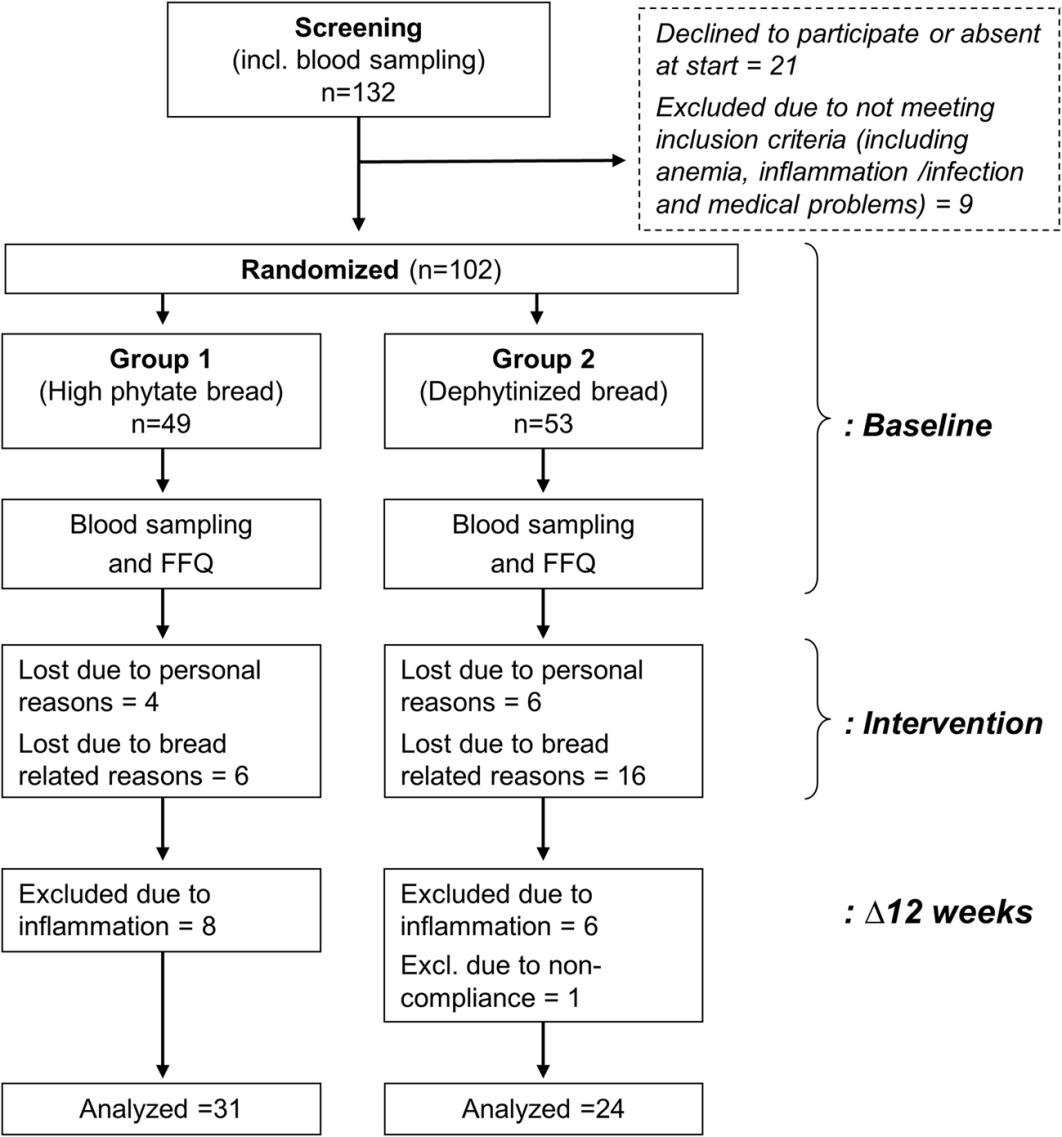
Study design. This was an intervention study over 12 weeks. After assessment of inclusion criteria and dietary habits, the subjects were allocated into two groups that either received (on a daily basis) 200 g wholegrain rye flour-based bread natural high in phytate, or 200 g dephytinized wholegrain rye flour-based bread. Evaluation was done at baseline and after 12 weeks by collection of blood samples and assessment of habitual diet

The study was a double-blind, randomized, parallel-design intervention study, which aimed to substitute part of the subjects’ habitual diet with 200 g wholegrain bread (mean ~ 74 g wholegrain/200 g bread) each day for a period of 12 weeks. This is close to the amount recommended per day by the Swedish Food Administration (75 g wholegrain/2000 kcal) [14]. Group 1 was administered wholegrain rye bread made from blanched rye and thus containing the natural amount of phytic acid (mean = 77 mg/200 g bread). Group 2 was administered dephytinized wholegrain rye bread (mean < 1.0 mg phytic acid/200 g bread). Habitual diet, height and weight were assessed, and non-fasting venous blood samples collected at baseline and after 12 weeks of intervention (Fig. 1). The administered bread was in addition to their habitual daily diet, and the subjects were encouraged to remain on their normal diet aside from substituting the bread. The study breads were self-collected by the subjects at the Department of Internal Medicine and Clinical Nutrition, Sahlgrenska Academy every other week.

### Randomized group allocation

A person not involved in the study organized randomization envelopes based on the throwing of a dice. Every time an uneven number appeared the person wrote an A on a card, and every time an even number appeared a B was written. The card was then put in an envelope consecutively numbered beginning from 1 and so on. For example, if the procedure generated the following series of letters: B, A, A, B, B, and so on, a card with the letter B was put in envelope 1, a card with the letter A was put in the next envelope, i.e. envelope 2, and in the third envelope a card with the letter A was put, and so on. When allocating a subject an envelope was drawn according to numerical order. In order to assign the subjects to the two groups the envelopes were then opened one at a time, in numerical order.

### Ethics

The study protocol, which was in accordance with the Helsinki Declaration of 1975 as revised in 1996, was approved by the regional ethical review board in Gothenburg (registration no. 520-05). Hence, the subjects were informed that they could withdraw from the study at any time without giving a reason.

### Subjects

Voluntary subjects of reproductive age (18–40 years) were recruited from the students attending the education programs (predominantly dietetics students) at the medical faculty (Sahlgrenska Academy) at the University of Gothenburg, and students at the Chalmers University of Technology. Women in this age range were selected as they are the main population at risk of iron deficiency. The subjects had to be females of reproductive age, healthy, non-smokers with no anemia, and should neither be pregnant nor lactating, nor exercising heavily. Medications or dietary supplements, including iron supplements, and blood donation less than two months before the start of the study were not allowed. Subjects had to have serum ferritin concentrations of > 15 µg/L and hemoglobin > 120 g/L. One hundred and thirty-two subjects were assessed for eligibility, of which 111 met the inclusion criteria (Fig. 1). After considering the exclusion criteria, nine subjects were excluded. Thus, 102 female subjects were included in the study; 49 and 53 subjects were randomized to the high-phytate intervention group and the low-phytate intervention group, respectively. All subjects were given written and oral information about the study, and were asked to sign a consent form.

### Dietary assessment

Dietary assessment was performed using a food frequency questionnaire (FFQ) used to measure habitual meal patterns and validated against doubly labeled water for total energy intake [15, 16]. In order to investigate habitual dietary patterns before and during the study, food containing dietary factors affecting iron absorption, such as coffee and tea, dairy products, citrus (fruit and/or juice), wholegrain rice, pasta or bulgur, meat, fish and poultry, were assessed. In the baseline FFQ they were asked to specify their habitual diet during the preceding 6 months, and in the post-intervention FFQ they were asked to specify their diet during the intervention period. All subjects also answered a post-intervention evaluation questionnaire in which they were asked how they experienced the bread, whether they had experienced any symptoms or side effects, if they had made any changes in their habitual diet, and if so, specify the changes. The definition of “servings/week” is number of times per week, regardless of portion size, that the particular foodstuff is consumed. Wholegrain bread was defined as containing ≥ 25% wholegrain content on a dry weight basis. The wholegrain content calculations in the FFQs were based on The Swedish Food Composition Database which provide information on the nutritional composition for more than 2000 foods and dishes [14]. Dietary phytate-P intake was calculated using the phytate data of Harland [17] and data obtained in our laboratory [8].

### Intervention

Subjects were instructed to incorporate five 40 g slices (total 200 g) of wholegrain bread/day for 12 weeks into their diet, equivalent to 74 g wholegrain rye. Subjects were encouraged to keep to their normal diet. The high-phytate bread group was given bread containing the natural amount of phytic acid (77 ± 11 mg/200 g bread). The low-phytate bread group was given bread based on sourdough, containing < 1.0 mg phytic acid/200 g bread.

### Intervention bread

In order to vary the bread eating, and by that increasing the compliance, two different types (A and B) of both the low-phytate and the high-phytate bread were alternated distributed to the subjects during this intervention. Besides water, yellow syrup, sodium chloride, yeast and liquid margarine, bread A was baked from wholegrain rye flour, wheat flour, bolted rye flour, and rye kernels. Bread B was baked from wholegrain wheat flour, wholegrain rye flour and wheat flour. These two breads were then baked with either phytate-degrading or phytate-preserving techniques, i.e. using scalded flour, which degrades phytases or by adding sourdough culture during the baking process. Different types of baking processes were investigated with the goal to select bread with a high wholegrain and fiber content and still palatable.

The bread-making procedure was first performed at the laboratory scale, and then scaled up for the trial. The total amount of bread needed was baked as different batches by a commercial bakery (Lantmännen Cerealia, Stockholm, Sweden) at their bakery in Lund, Sweden and transported fresh to the Swedish Institute for Food and Biotechnology in Gothenburg, where it was stored in large freezers (− 20 °C), awaiting delivery to the subjects. Before administering the bread each batch was analyzed for phytate and iron content (see below).

Phytate removal of the low-phytate bread was performed by food processing at optimal conditions for phytate-degrading enzymes. Dephytinized of whole kernels produced by hydrothermal treatment or soaking at optimal conditions previously shown to remove 95% of the phytate in barley kernels [18, 19]. Breads made by mild scalding (70C, 4 h, added lactic acid) of rye bran added to a wheat dough or made with 10% sourdough was previously demonstrated to reduce the phytate content by 96–97% [20]. We therefore used addition of 10% sourdough to the baking dough to produce breads for the intervention. The sourdough fermentation was performed during 24 h. Both high and low-phytate breads were fermented for 50 min at 37° and baked at 200 °C for 55 min.

Each daily ration of wholegrain bread (200 g) contained 2.4 mg non-heme Fe, 2.5 mg ascorbic acid, 10 mg Ca and contained 40% wholegrain. The content of phytic acid in the bread administered to the high-phytate bread group was 77 ± 11 mg/200 g bread (mean ± SD). The mean phytic acid content in the dephytinized low-phytate bread was < 1 mg/200 g bread.

Phytate and its degradation products were determined by a method developed at the Department of Chemical and Biological Engineering, Food and Nutrition Science, Chalmers University of Technology, Gothenburg, Sweden, using high performance ion chromatography [21]. The mineral content of the breads was analyzed by HPLC coupled with UV–Vis detection [22].

### Organic acid analysis/assay

Organic acids in breads were determined with HPLC according to a modification of the method of Ashoor and Knox [23] as described in Scheers et al. [24].

### Compliance

Apart from eating the bread, all subjects were asked to maintain their habitual dietary patterns for the duration of the study. Every week the subjects came to our laboratory for collecting their weekly ration of bread. At these occasions compliance and possible problems or discomfort due to the bread was evaluated by face-to-face interviews with the subjects. If experiencing any problems in-between these occasions it was emphasized to the subjects to contact the research team. Compliance was also assessed using plasma alkylresorcinols (see below for the method used), a non-biased measure that reflects wholegrain wheat and rye intake [25].

### Anthropometric and laboratory measurements

Weight and height were measured with the subjects in light clothes and no shoes. Blood samples were collected at baseline as well as after 12 weeks of intervention by placing an intravenous catheter in a peripheral arm vein. The following blood samples were collected: Hemoglobin concentration (Hb), serum iron concentration (S-Fe), total iron binding capacity (TIBC), transferrin saturation (TSAT), serum ferritin concentration (SF), and soluble transferrin receptor (sTfR). The presence of infection/inflammation [26] was checked by analyzing for the acute-phase proteins C-reactive protein (CRP) and alpha 1-acid glycoprotein (AGP), as infection and inflammation can stimulate the production of ferritin without being related to iron status. The analyses were conducted at an accredited reference laboratory (Clinical Chemistry Laboratory, Sahlgrenska University Hospital, Gothenburg, Sweden), according to ISO/IEC 15 189 Standard for Medical Laboratories. Primary outcome variables were SF, sTfR, Hb, and change in amount of body iron reserves according to Cook et al. [27], which is based on the ratio of soluble transferrin receptor to serum ferritin. Serum hepcidin concentration at base line and post intervention was determined using a peptide enzyme immunoassay (Peninsula Laboratories, LLC, San Carlos, CA, USA).

### Plasma alkylresorcinols

Blood for plasma alkylresorcinol analysis were drawn in a non-fasting state. Plasma alkylresorcinols were measured using normal-phase liquid chromatography coupled to tandem mass spectrometry (LC-MS/MS), after extraction from EDTA-plasma using supported liquid extraction (SLE) [28]. Briefly, 20 µL of 10 ng/mL internal standard (alkylresorcinol C19:0 d4, Reseachem AG, Bergsdorf, Switzerland) was added to 100 µL of plasma, and the sample extracted twice with 800 µL acetone on a SLE plate (HybridSPE, Sigma-Aldrich). Pooled extract was dried and resuspended in 50 µL of heptane:ethanol (95:5 v/v) and 10 µL injected onto an LC-MS/MS system (Shimadzu LCMS 8040, Shimadzu Europa GmbH, Duisburg, Germany). Alkylresorcinol homologues C17:0, C19:0, C21:0, C23:0 and C25:0 were quantified relative to the internal standard. Batch quality control samples had inter- and intra-batch variation < 15%.

### Laboratory measurements for infection

A previous WHO/CDC expert consultation on assessing iron status of populations suggested that together with the iron status measurements at least one laboratory measurement for infection (such as CRP and AGP) should be analyzed [29] to control for bias introduced by infection/inflammation. In addition, at blood sampling the subjects were asked about signs of infection or inflammation, such as a cold, cough, sore throat, or fever during the previous weeks. Positive answers regarding infections and/or CRP > 5 mg/L and/or AGP > 1.2 g/L were considered to be exclusion criteria.

### Power calculation

The primary hypothesis was that after 12 weeks of a daily intake of 200 g wholegrain rye bread with very low amounts of phytate would improve markers of iron status compared to wholegrain rye bread with high amounts of phytate. Approximately 30 subjects are needed in order to have an 85% probability (i.e. a power of 85%) to observe a 5 unit increase in S-ferritin from an initial 30 µg/L at an SD of 9, when a significant effect actually exists. With an expected 15% drop-off frequency a total of 35 subjects per group are needed.

### Statistical analyses

Data are mainly presented as geometric means and standard error of the mean (SEM). However, data including zero or negative values are presented as median and interquartile range (IQR). Test for normality was done using a Shapiro–Wilk test. Data fitting the Gaussian distribution were analyzed by paired-sample *t* tests when analyzing changes over time within groups, and independent samples *t* test was used when analyzing changes over time between groups. Wilcoxon signed-rank test and Mann–Whitney *U* test were applied for non-normally distributed values. All *p* values are two-tailed and considered to be statistically significant if < 0.05. The statistics program used was SPSS for Windows version 22.0.0.0 (SPSS Inc., Chicago, IL, USA).

## Results

### Baseline

One hundred and two subjects were included for randomization into the study. At baseline, the high-phytate bread group consisted of 49 subjects, and the low-phytate bread group consisted of 53 subjects (Fig. 1).

### Withdrawals and exclusions

Flow diagram of the progress through the phases of the trial, including withdrawals (31%) and exclusions (14%), are shown in Fig. 1. During the intervention in the low-phytate bread group 6 subjects were lost due to personal reasons, and another 16 were lost due to bread related reasons. In the high-phytate bread group four subjects were lost due to personal reasons, and six were lost due to bread related reasons. When analyzing the data post-intervention, eight were excluded due to inflammation in the high-phytate bread group, and six were excluded due to inflammation in the low-phytate bread group. One subject in the low-phytate bread group was excluded due to non-compliance regarding eating the intervention bread. In total, out of the 102 subjects starting, 32 withdrew from the study during the intervention, giving a drop-out rate of 31%; 70 subjects completed the 12 week intervention, of which 15 subjects were excluded, of which 1 who reported eating only half of the required amount of bread. The number of subjects included in the final analysis was 24 and 31 for the sourdough (low-phytate) bread group and the high-phytate bread group, respectively (total *n* = 55). Geometric mean age in the high-phytate bread group and the low-phytate bread group was 25.7 ± 4.6 years (*n* = 31) and 25.4 ± 5.2 years (*n* = 24), respectively. Four of the subjects in the high-phytate bread group, and one subject in the low-phytate bread group were vegetarians. There were no significant differences in any of the baseline anthropometric and laboratory values (Table 1) between the two groups.

**Table 1:** Data at baseline and after 12 weeks of intervention

	High-phytate bread group (*n* = 31)			Low-phytate bread group (*n* = 24)			
	Baseline	Post-intervention	*p* value (within group)	Baseline	Post-intervention	*p* value (within group)	*p* value (between groups)
BMI (kg/m^2^)	22.6 ± 0.6	22.8 ± 0.6	NS	21.7 ± 0.5	21.8 ± 0.5	NS	NS
Hb (g/L)	134 ± 1	136 ± 1	NS	132 ± 1	134 ± 1	NS	NS
Hepcidin (ng/ml)	11.8 ± 2.4	11.8 ± 2.0	NS	13.6 ± 2.7	9.5 ± 2.8	NS	NS
Ferritin (µg/L)	31 ± 4	32 ± 4	NS	33 ± 3	27 ± 6	**0.018**	NS
TfR (mg/L)	2.7 ± 0.1	2.7 ± 0.1	NS	2.9 ± 0.1	2.9 ± 0.6	NS	NS
Body Fe^a^ (mg/Kg)	7.0 ± 0.4	7.0 ± 0.5	NS	6.9 ± 0.4	5.4 ± 0.5	**0.035**	NS
Alkyl-resorcinols (mmol/L)	351 ± 52	467 ± 77	NS	266 ± 68	522 ± 127	**0.002**	NS
C17:C21 ratio	0.16 ± 0.02	0.23 ± 0.02	**0.018**	0.15 ± 0.02	0.29 ± 0.03	**0.001**	0.051

Subjects were allocated into two groups that either received (on a daily basis) 200 g wholegrain rye flour-based bread natural high in phytates or 200 g dephytinized wholegrain rye flour-based bread. Evaluation was done at baseline and after 12 weeks. Values represent geometric mean ± standard error of the mean (SEM)

Bold values indicate the level of significance *p* ≤ 0.05

^a^Calculation of body iron reserves was based on the ratio between soluble transferrin receptor and serum ferritin [27]

### Dietary changes during the intervention

In the high-phytate bread group, 18 stated that they had exclusively replaced carbohydrate-based meals with the intervention bread. None had exclusively replaced cooked meals. Nine had replaced both cooked meals and carbohydrate-based meals. Three had not replaced anything in their diets, i.e., they stated that they had put the administered intervention bread on the top of their habitual diet. One did not answer.

In the low-phytate bread group, 12 stated that they had exclusively replaced carbohydrate-based meals with the intervention bread. None had exclusively replaced cooked meals. Twelve had replaced both cooked meals and replaced carbohydrate-based meals.

### Post-intervention iron status

Of all the studied iron biomarkers, the only observed changes were that S-ferritin (*p* < 0.018) and amount of body iron reserves (*p* < 0.035) decreased within the low-phytate bread group following 12 weeks of intervention. Within the high-phytate bread group, no changes were observed (*p* > 0.05). Neither were there any differences in changes over time between the two groups (Table 1).

### Wholegrain intake

The reported median number of slices of wholegrain bread consumed per day at baseline was 2.6 [IQR = 1.7] and 2.5 [IQR = 1.9] slices/day for the subjects in the high-phytate bread group (*n* = 31) and the low-phytate bread group (*n* = 24) respectively (Table 2). During the study this increased to 5.0 [IQR = 0.0] slices/day for both the high-phytate bread and low-phytate bread groups (*p* = 0.001 for both comparing baseline vs. intervention).

**Table 2:** Whole grain intake at baseline and after 12 weeks of intervention

	High-phytate bread group (*n* = 31)			Low-phytate bread group (*n* = 24)			
	Baseline	During intervention	*p* value^3^ (within group)	Baseline	During intervention	*p* value (within group)	*p* value (between groups)
Total WG intake (g/day)	75 ± 47	111 ± 27	**0.010**	81 ± 51	112 ± 35	0.076	NS
WG intake in connection with main meals (g/day)	69 ± 43	90 ± 34	**0.046**	76 ± 49	93 ± 41	NS	NS
WG intake when excluding WG from bread (g/day)	42 ± 28	31 ± 28	**0.014**	43 ± 39	34 ± 34	**0.003**	NS
WG intake from total dietary bread (g/day)	27 ± 25	76 ± 6	**0.001**	30 ± 23	76 ± 6	**0.001**	NS
WG intake from bread alone in connection with main meals (g/day)	23 ± 21	55 ± 19	**0.001**	24 ± 23	55 ± 18	**0.001**	NS

Data are presented as geometric mean ± standard error of the mean (SEM)

Bold values indicate the level of significance *p* ≤ 0.05

### Phytate-P intake

**Fig. 2:**
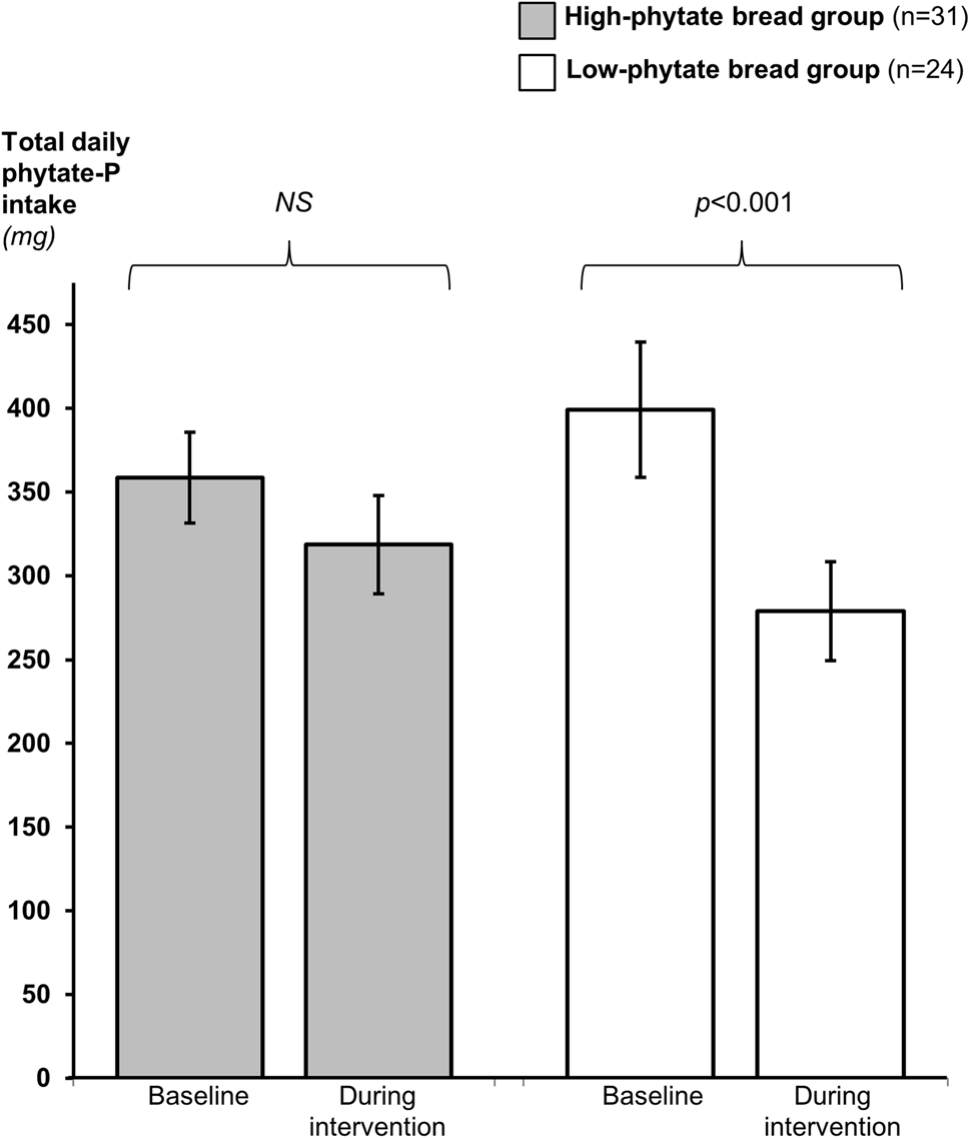
Dietary phytate-P intake. Data are presented as geometric mean ± standard error of the mean (SEM)

The total daily dietary phytate-P intakes at baseline in the two groups were not different (*p* > 0.05) (Fig. 2). During the intervention the low-phytate bread group decreased their total dietary phytate-P intake from 399 ± 40 to 279 ± 29 mg/day (*p* < 0.001), whereas there was no change in the high-phytate bread group (baseline: 358 ± 27 mg/day; during the study: 318 ± 29 mg/day). The change in phytate-P intake over time was greater in the low-phytate bread group (median = − 114 mg/day) than in the high-phytate bread group (median = − 63 mg/day, *p* = 0.021).

### Organic acids

The phytate degradation processes used in the development of the dephytinized wholegrain bread had resulted in changes in organic acid concentrations (Table 3).

**Table 3:** Bread content

	(200 mg)		(200 mg)	
	Mean	Sd	Mean	Sd
Iron (mg)	2.5	0.6	2.4	0.2
Phytate-P (mg)	22	3	< 1	–
Lactic acid (mg)	12	7	2300	168
Citric acid (mg)	29	4	ND	–
Succinic acid (mg)	860	188	207	43
Acetic acid (mg)	ND	–	225	27
Calcium (mg)	10	–	10	–
Ascorbic acid (mg)	3	–	3	–
pH	4.6	–	4.0	–

1 mg Phytate phosphorus = 3.5 mg, phytic acid = 5.56 µmol phytic acid

*Phytate-P* phytate phosphorus, *ND* not detectable

### Inflammation markers

At the post-intervention blood sampling four subjects in the high-phytate bread group had CRP > 5 mg/L, and another four subjects gave positive answers regarding signs of infection or inflammation during the previous weeks. In the low-phytate bread group three subjects had CRP > 5 mg/L, and another three subjects in the high-phytate bread group gave positive answers regarding signs of infection or inflammation. None had elevated AGP concentrations.

### Dietary habits

**Fig. 3:**
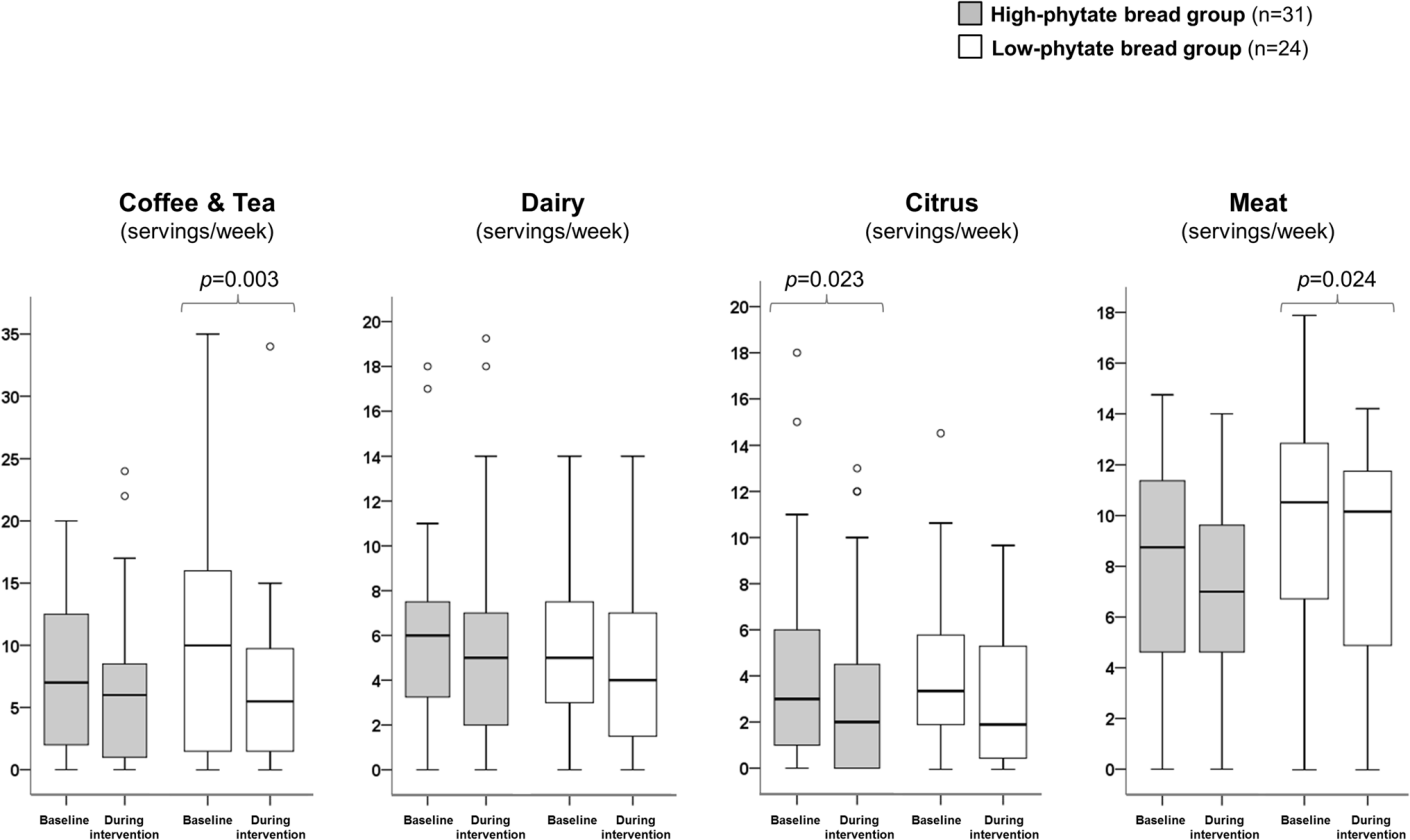
Dietary intake. The figures illustrates intake in connection with main meals, i.e., breakfast, lunch, and dinner. The definition of “servings/week” is number of times per week, regardless of portion size, that the particular food stuff is consumed. Data are illustrated as box plots covering the lower to the upper quartile (i.e., the 25th to the 75th percentile). The band inside the box depicts the median value (the 50th percentile) and the whiskers depict values up to 1.5 times the interquartile range (IQR). Values greater than 1.5 times the IQR but less than 3 times the IQR (from the end of the box) are labeled as outliers (O). Values greater than three times the IQR (from the end of a box) are labeled as extreme (indicated by an asterisk)

In the high-phytate bread group (*n* = 31) there were within-group changes in the frequency that citrus (fruit and/or juice) had been eaten and/or drunk in connection with main meals, i.e., breakfast, lunch, and dinner (baseline: 3.0 times/week [IQR = 5.0] vs. during: 2.0 times/week [IQR = 5.0], *p* = 0.023) (Fig. 3).

The change in the frequency of coffee and tea intake in connection with main meals (7.0 times/week [IQR = 12.0]) at baseline vs. during the intervention (6.0 times/week [IQR = 8.0]) in the high-phytate bread group was borderline significant (*p* = 0.054).

In the low phytate bread group (*n* = 24) there were significant within-group changes in frequency of: (1) coffee and tea intake in connection with main meals, i.e., breakfast, lunch, and dinner (10.0 times/week [IQR = 15.0]) at baseline vs. during the intervention (5.5 times/week [IQR = 9.0], *p* = 0.003); and (2) meat, fish and/or poultry was eaten in connection with lunch or dinner (baseline: 10.8 times/week [IQR = 6.0] vs. during: 10.4 times/week [IQR = 15.0], *p* = 0.017). The difference in the frequency of citrus intake in connection with main meals in the low-phytate bread group (*n* = 24) between baseline and the intervention was borderline significant (baseline: 3.5 times/week [IQR = 4.0] vs. during: 2.0 times/week [IQR = 6.0], *p* = 0.053).

### Plasma alkylresorcinols

**Fig. 4:**
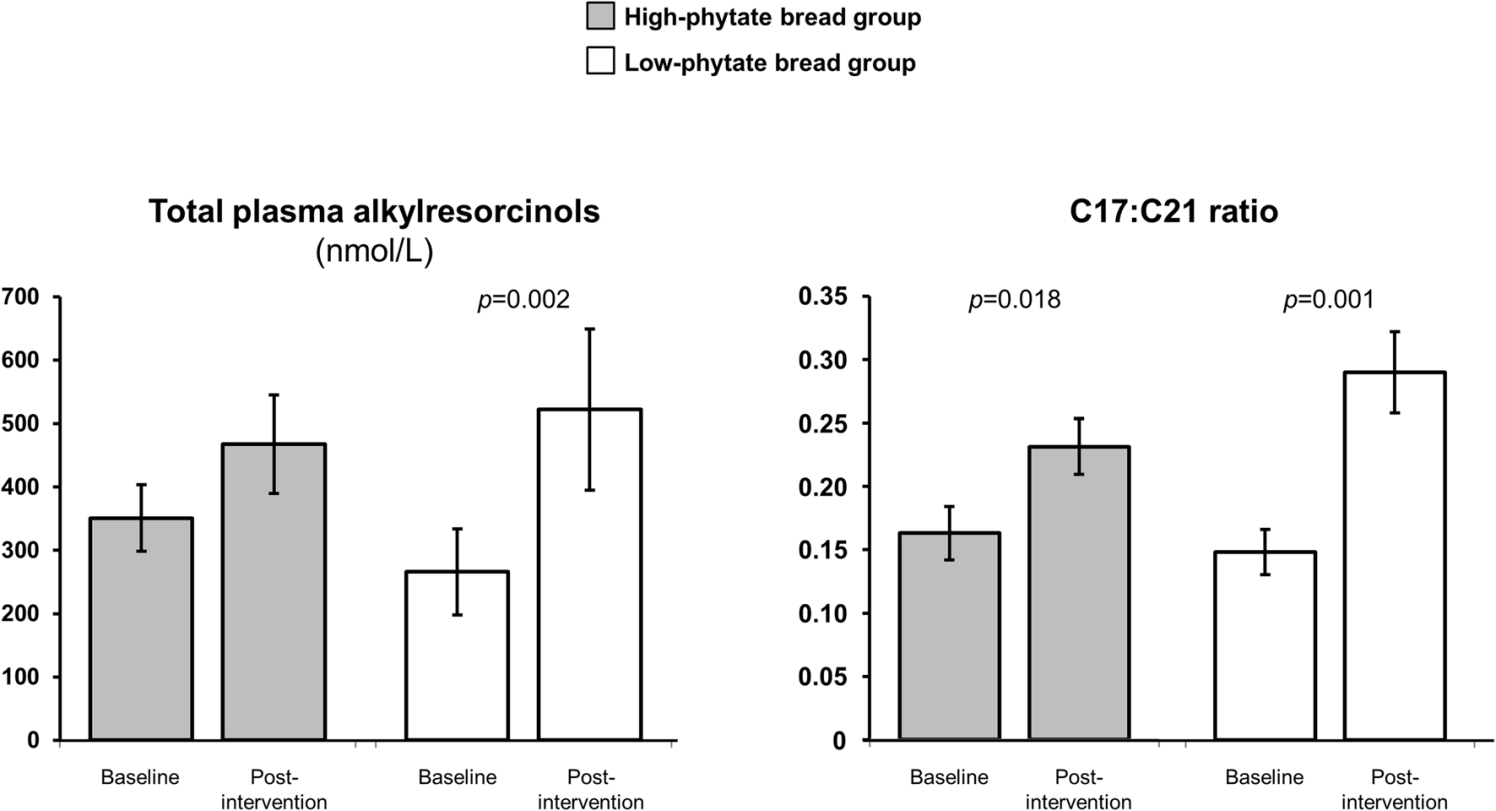
Alkylresorcinols. Total plasma alkylresorcinols and ratio of alkylresorcinol homologues C17:C21 in the high-phytate bread group and the dephytinized wholegrain bread group. The ratio of alkylresorcinol homologues C17:C21 indicates the proportion of wheat and rye in the diet. Data are presented as geometric mean ± standard error of the mean (SEM)

Non-fasting plasma alkylresorcinol concentrations were 351 ± 52 nmol/L for the high-phytate bread group and 266 ± 68 nmol/L (geometric mean ± SD) for the low-phytate bread group at baseline, with samples not available for one subject in the high-phytate bread group and two in the low-phytate bread group (Fig. 4). After the 12 week intervention, plasma alkylresorcinols increased to 467 ± 77 and 522 ± 127 nmol/L for the high-phytate and low-phytate bread groups, respectively. The low-phytate bread group increased plasma alkylresorcinol concentrations from baseline (*p* < 0.002). The plasma alkylresorcinol concentrations did not differ between the interventions at either baseline or 12 weeks.

The C17/C21 ratio, an increase of which reflects an increase in the relative proportion of rye vs. wheat intake [30] increased after the 12 week intervention from 0.16 ± 0.02 and 0.15 ± 0.02 at baseline to 0.23 ± 0.02 and 0.29 ± 0.03 after 12 weeks for the high-phytate and the low-phytate bread groups, respectively (*p* = 0.018 and *p* = 0.001 for difference between baseline and 12 weeks for high-phytate and low-phytate bread groups, respectively). These changes reflect a greater intake of wholegrain rye during the intervention. There was no difference between the diet intervention groups at baseline, although a tendency for a higher C17/C21 ratio at 12 weeks in the low-phytate bread group (*p* = 0.08).

Four subjects (two from each group) had very low plasma alkylresorcinol concentrations after the intervention that would suggest that they may not have complied fully with the study diet based on a conservative threshold of < 130 nmol/L estimated from earlier dose response studies based on fasting plasma [31]. As plasma alkylresorcinols were measured in non-fasting plasma, mean concentrations are considerably higher than those reported in other intervention studies using fasting plasma.

### Correlation analyses

In the high-phytate bread group there was an inverse relationship between intake of meat (incl., fish and poultry) during the study and sTfR post-intervention (*r* = − 0.381, *p* = 0.034, *n* = 31).

In the low-phytate bread group there was an inverse relationship between logarithmic transformed change in S-ferritin from baseline to post-intervention and total wholegrain intake (incl., wholegrain intake study bread) from mean meals and wholegrain intake from the study bread during the intervention (*r* = − 0.415, *p* = 0.044, and *r* = − 0.478, *p* = 0.018, respectively).

## Discussion

The primary hypothesis of this study was that iron status in women would be improved by providing bread with negligible amounts of phytate in comparison with standard wholegrain bread contributing a high amount of phytate to the diet. Surprisingly, we found that the results contradicted this hypothesis. When analyzing dietary habits it was observed that the total wholegrain intake at baseline was high in both groups, and at or above the minimum suggested by the Nordic Nutrition Recommendations (75 g/2000 kcal) [14, 32]. Consequently, also the phytate-P at baseline was high (> 350 mg/day). For comparison, non-lactating young women in the United States consume 113 mg/day phytate-P, while the phytate-P intake in ovo-lacto-vegetarian Trappist monks in the United States was 1305 mg/day. In the United Kingdom the phytate-P intake has been reported to be 100–200 mg/day [33]. For the purposes of inhibition of metal uptake, it has been shown that it is the first 10–20 mg phytate-P in a meal exerts the largest inhibitory effect, and that additionally increase of phytate-P in the same meal has a relatively minor effect [8, 9, 13]. Thus, a nearly complete elimination of phytate content is necessary to have any effect on iron bioavailability. Phytates coming from other parts of the subjects’ habitual diet may have nullified any effect of the low-phytate bread on iron bioavailability. Similar to our findings, an 8 week high-phytate diet had no effect on ferritin, transferrin receptor, and hepcidin concentrations in 28 females [34].

In the low-phytate bread group there was an unexpected decrease in markers of iron status. We had hypothesized that the low-phytate intervention bread would increase the availability of iron leading to an increase in iron status. However, an explanation for the opposite effect is not immediately clear. The total wholegrain intake did not change during the study. However, when excluding the wholegrain contributed by bread, the remaining wholegrain intake actually decreased from 43 g/day at baseline to 34 g/day in the low-phytate bread group. Also total daily phytate-P intake decreased during the study from 399 to 279 mg/day. This together with the decrease in number of coffee and tea servings per week would speak in favor of an overall positive effect on iron status in the low-phytate bread group. However, since the servings of meat decreased, along with a tendency towards a decrease in citrus (i.e., vitamin C) servings per week, this could have had a negative effect on iron bioavailability. A decrease in meat intake does not only result in a reduction of the so-called meat factor [35–37], but also a decrease in a valuable source of both heme iron and non-heme iron.

In the high-phytate bread group, although not significant, there were tendencies for decreased intake of meat, dairy, coffee and tea. Previously an intervention showed that 120 g wholegrain/day led to a decrease in fruit and vegetable intake, although intake of 60 g wholegrain did not [38]. This suggests that in a habitual diet situation where subjects may replace, add, or both replace and add the study food, the displacement of other foods important for iron intake may be a major confounding factor that offset any improvement of iron uptake from the low-phytate bread. In this present study no effects were seen on body weight for either group, which along with the high rate of compliance, suggests that subjects were mainly replacing other foods with the study breads.

Follow-up analyses of the bread used in the intervention showed that the phytate degradation processes used in the development of the dephytinized wholegrain bread also had given rise to high lactic acid concentrations. Although lactic acid has been suggested to have an enhancing effect on iron absorption [39, 40], in vitro studies suggest that high levels of lactic acid may cause a dose-dependent reduction of iron uptake [41]. However, when we performed further follow-up analyses of the intervention bread in Caco-2 models the iron uptake was significantly higher in the dephytinized (and thus lactic acid loaded) wholegrain bread (unpublished data). This is in line with previous studies that lactic acid fermentation both increases soluble iron content and stimulates iron absorption in Caco-2 cells [42]. These results suggest that the high amount of lactic acid may not have been responsible for a decrease in iron uptake from the bread. On the other hand, since high amounts of lactic acid result in an acidic taste, other explanations for the reduction in iron status after eating the low-phytate bread could perhaps include taste, since this may have altered the way that subjects ate the bread. No data were collected on how they ate the bread, i.e., what they ate or/and drank together with the bread.

The plasma alkylresorcinol results affirm the FFQ results showing that the subjects were consuming diets very high in wholegrain. As expected, mean concentrations were also further increased after the intervention. The high baseline wholegrain intake adds a further confounder in this study, as it can be questioned whether adding more iron from either wholegrain rye bread or sourdough rye bread, irrespective of the phytate content, would have any substantial impact on iron status. The tendency for an increase in alkylresorcinol C17:C21 ratio suggests that the subjects ate proportionally more rye bread during the intervention, supporting the FFQ-based data that subjects complied with the intervention.

In older subjects in the Framingham Offspring Study, increased wholegrain intake was associated with reduced iron status [43]. In this present study, intakes of > 100 g wholegrain did not lead to any change in iron status, albeit from a baseline where subjects were already consuming a high amount of wholegrain. Thus, this present study demonstrates that although phytate reduction per se has proven to be an effective measure to enhance iron bioavailability, this measure is not enough if the iron bioavailability of the remaining diet tip the balance scale towards the negative side.

Although the dietary phytate load in this study was greatly reduced using the low-phytate bread, it is impossible to rule out that other sources of phytate may have been sufficient to mitigate any improvement in iron intake, given the ability of small amounts of phytate to bind large amounts of iron. A further limitation is the reliance of a FFQ to determine phytate intake, which relies on database values for phytate, which are known to vary widely due to agricultural factors, etc. [44, 45], though such limitations are inherent in all studies on phytochemicals in food. Future studies looking at the long-term benefits should also use more controlled diets, though such studies are more expensive and demanding on the subjects. A controlled diet would also allow for better estimation of phytate intake with each meal, so that other sources of phytate that may negate the added availability of iron from low-phytate bread could be accounted for. The selected population may also have an impact on the outcomes as the subjects were healthy and observed to be ‘health conscious’. Selecting other groups with a low overall iron status or poor overall iron availability in their diet may have led to different incomes if iron bioavailability becomes a major determinant in overall iron status.

There was a high dropout rate, and as such it may have affected the results. Especially when using the presently used approach, per-protocol (PP) analysis, since it can give an overestimation of the treatment effect. An alternative approach would be intent-to-treat (ITT). A problem with ITT may be that the subjects are mixed up with individuals who have not received the intended intervention at all, which gives a dilution effect and an underestimation of the effect in subjects who are really exposed to the intervention. However, when performing an ITT analysis on our data by using the principle of “last observation carried forward” the result was similar to the PP analysis. There was a tendency for a decrease in ferritin (*p* < 0.053) and a decrease in total body iron (*p* < 0.039) in the low phytate bread group. Also, due to the relatively high number of subjects who did not complete the study, the use of the PP results is more reliable.

In conclusion, although single-meal efficacy studies clearly show an increase in iron bioavailability from dephytinization of plant sources of iron, medium-term consumption of phytate-reduced bread under free-living conditions suggests that this strategy alone is not sufficient for improving iron status in healthy women of reproductive age. Overall plausible explanations for the present findings could be that the subjects altered their diet in a way that shifted the balance between various inhibitors and enhancers of iron intake. Also, in the context of a mixed diet, adding additional wholegrain to a diet already high in wholegrain does not appear to lead to any decrease in iron status in healthy young women.
