# Correction to: Low-phytate wholegrain bread instead of high-phytate wholegrain bread in a total diet context did not improve iron status of healthy Swedish females: a 12 week, randomized, parallel-design intervention study

**DOI:** 10.1007/s00394-020-02286-1

**Published:** 2020-07-09

**Authors:** Michael Hoppe, Alastair B. Ross, Cecilia Svelander, Ann‑Sofie Sandberg, Lena Hulthén

**Affiliations:** 1 Department of Gastroenterology and Hepatology, Clinical Nutrition Unit, Sahlgrenska University Hospital, Göteborg, Sweden; 2 Department of Internal Medicine and Clinical Nutrition, Sahlgrenska Academy at the University of Gothenburg, Göteborg, Sweden; 3 Department of Biology and Biological Engineering, Food and Nutrition Science, Chalmers University of Technology, Göteborg, Sweden

## Correction to: European Journal of Nutrition (2019) 58:853–864 https://doi.org/10.1007/s00394-018-1722-1

Due to author error the paper was published with a statistical fault.

The “Results” section in the abstract should read: “Fifty-five females completed the study. There was a significant difference in change over time in total body iron stores between the two groups (*p* < 0.035). In the low-phytate bread group (*n* = 24) there were significant within-group decreases in both ferritin (mean 12%; from 32 ± 7 to 27 ± 6 μg/L, geometric mean ± SEM, *p* < 0.018) and total body iron (mean 12%; from 6.9 ± 1.4 to 5.4 ± 1.1 mg/kg, *p* < 0.035). Plasma alkylresorcinols indicated that most subjects complied with the intervention”.

The first sentence in the “Conclusions” section in the abstract should read: “In Swedish females of reproductive age, no statistically significant difference in iron status was detected after 12 weeks of high-phytate wholegrain bread consumption”.

The “Post-intervention iron status” Result section should read: “Following 12 weeks of intervention there was a significant difference in change over time in total body iron stores between the two groups (*p* < 0.035, Table 1). Within the low-phytate bread group there were a decrease in S-ferritin (*p* < 0.018) and amount of body iron reserves (*p* < 0.035).”

**Table 1:** Data at baseline and after 12 weeks of intervention

	High-phytate bread group (*n* = 31)			Low-phytate bread group (*n* = 24)			*p* value (between groups)
	Baseline	Post-intervention	*p* value (within group)	Baseline	Post-intervention	*p* value (within group)	
BMI (kg/m^2^)	22.6 ± *0.6*	22.8 ± *0.6*	0.326	21.7 ± *0.5*	21.8 ± *0.5*	0.145	0.774
Hb (g/L)	134 ± *1*	136 ± *1*	0.155	132 ± *1*	134 ± *1*	0.178	0.682
Hepcidin (ng/mL)	11.8 ± *2.4*	11.8 ± *2.0*	0.704	13.6 ± *2.7*	9.5 ± *2.8*	0.433	0.343
Ferritin (µg/L)	31 ± *4*	32 ± *4*	0.859	33 ± *3*	27 ± *6*	**0.018**	0.251
TfR (mg/L)	2.7 ± *0.1*	2.7 ± *0.1*	0.409	2.9 ± *0.1*	2.9 ± *0.6*	0.489	0.491
Body Fe^a^ (mg/kg)	7.0 ± *0.4*	7.0 ± *0.5*	0.738	6.9 ± *0.4*	5.4 ± *0.5*	**0.035**	**0.035**
Alkyl-resorcinols (mmol/L)	351 ± *52*	467 ± *77*	0.120	266 ± *68*	522 ± *127*	**0.002**	0.139
C17:C21 ratio	0.16 ± *0.02*	0.23 ± *0.02*	**0.018**	0.15 ± *0.02*	0.29 ± *0.03*	**0.001**	0.051

Subjects were allocated into two groups that either received (on a daily basis) 200 g wholegrain rye flour-based bread natural high in phytates or 200 g dephytinized wholegrain rye flour-based bread. Evaluation was done at baseline and after 12 weeks

Values represent geometric mean ± standard error of the mean (SEM, as italicized values)

Bold values indicate the level of significance *p* ≤ 0.05

^a^Calculation of body iron reserves was based on the ratio between soluble transferrin receptor and serum ferritin [27]

Also, Table 1 and 2 should state the actual *p* values, and not just NS (not significant). Thus, revised tables are provided. Accordingly, the *p* value for change over time in dietary phytate-P intake for the high-phytate bread group in Fig. 2 should state “*p* = 0.106”.

**Table 2:** Whole grain intake at baseline and after 12 weeks of intervention

	High-phytate bread group (*n* = 31)			Low-phytate bread group (*n* = 24)			*p* value (between groups)
	Baseline	During intervention	*p* value^3^ (within group)	Baseline	During intervention	*p* value (within group)	
Total WG intake (g/day)	75 ± *47*	111 ± *27*	**0.010**	81 ± *51*	112 ± *35*	0.076	0.696
WG intake in connection with main meals (g/day)	69 ± *43*	90 ± *34*	**0.046**	76 ± *49*	93 ± *41*	0.263	0.622
WG intake when excluding WG from bread (g/day)	42 ± *28*	31 ± *28*	**0.014**	43 ± *39*	34 ± *34*	**0.003**	0.350
WG intake from total dietary bread (g/day)	27 ± *25*	76 ± *6*	**0.001**	30 ± *23*	76 ± *6*	**0.001**	0.760
WG intake from bread alone in connection with main meals (g/day)	23 ± *21*	55 ± *19*	**0.001**	24 ± *23*	55 ± *18*	**0.001**	0.629

Data are presented as geometric mean ± standard error of the mean (SEM, as italicized values)

Bold values indicate the level of significance *p* ≤ 0.05

